# Integrated multi-omics analysis reveals rumen and rectal microbiota–metabolite interaction features in polytocous fine-wool sheep with divergent residual feed intake

**DOI:** 10.3389/fmicb.2025.1712307

**Published:** 2026-01-22

**Authors:** Menghua Kong, Zhangyuan Pan, Xu Wang, Juncheng Huang, Hanikezi Tulafu, Yue Xu, Yiming Sulaiman, Weiwei Wu

**Affiliations:** 1 College of Animal Science, Xinjiang Agricultural University, Urumqi, China; 2 Animal Husbandry Research Institute, Xinjiang Academy of Animal Husbandry Sciences, Urumqi, China; 3 State Key Laboratory of Animal Biotech Breeding, Institute of Animal Science, Chinese Academy of Agricultural Sciences (CAAS), Beijing, China; 4 College of Animal Science and Technology, Tarim University, Alar, China

**Keywords:** gut microbiota, metabolomics, metagenomics, residual feed intake, rumen

## Abstract

Residual feed intake (RFI) is a key indicator of feed efficiency in ruminants. To elucidate the potential regulatory roles of microorganisms and metabolites under different RFI levels, we investigated 24 polytocous fine-wool sheep (12 high-RFI and 12 low-RFI) using metagenomic sequencing and non-targeted metabolomics of rumen and rectal contents. Significant differences in average daily feed intake, residual feed intake, and feed conversion ratio were observed between groups (*p* < 0.001). LEfSe analysis identified four and seventeen RFI-associated microbial biomarkers in the rumen and rectum, respectively, with *s_Ruminococcus_albus* and *s_Ruminococcus_bicirculans* as common core taxa. Functional annotation revealed that high-RFI sheep were enriched in amino acid metabolism and xenobiotic degradation pathways in the rumen, whereas low-RFI sheep were enriched in pathways related to development and regeneration. In the rectum, high-RFI sheep showed enrichment in protein folding and degradation, carbohydrate metabolism, and energy metabolism, while low-RFI sheep were enriched in transcriptional regulation and signal transduction pathways. Metabolomic analysis detected 297 and 1,130 differential metabolites in the rumen and rectum, respectively, mainly lipids, organic acids, and derivatives. KEGG enrichment indicated that rumen metabolites were primarily involved in bile acid biosynthesis and riboflavin metabolism, while rectal metabolites were enriched in energy metabolism and multiple amino acid pathways, including arachidonic acid, tryptophan, tyrosine, lysine, and methionine metabolism. Integrated analysis revealed significant associations between key bacterial taxa and metabolites, and network construction identified core nodes potentially engaged in synergistic regulation, providing insights into their roles in RFI phenotype formation. Collectively, these findings highlight the distinct contributions of the rumen and rectum to feed efficiency in sheep and offer theoretical support for nutritional regulation strategies to improve ruminant production performance.

## 1 Introduction

The polytocous fine-wool sheep is a newly developed breed produced by crossing Brulla Merino rams with Chinese Merino ewes (military reclamation type). This breed combines high reproductive performance with superior wool quality, making it an important genetic resource for enhancing the economic efficiency of sheep production (Wang et al., 2024). However, with the expansion of farming operations, feed costs have risen to account for more than 60% of the total production costs in ruminants, becoming a major constraint to profitability (Si et al., 2020). Improving feed utilization efficiency has therefore emerged as one of the core objectives for the sustainable development of the livestock industry. Residual feed intake (RFI) is a key metric for evaluating feed efficiency, defined as the difference between an animal’s actual feed intake over a given period and its predicted feed intake based on maintenance and growth requirements (Koch et al., 1963). RFI is negatively correlated with feed efficiency; animals with lower RFI values consume less feed while maintaining the same growth rate, thereby achieving higher feed conversion efficiency (Zhang et al., 2017). Compared with the traditional feed conversion ratio (FCR), RFI more accurately reflects individual differences in metabolic efficiency and exhibits moderate heritability and high repeatability (Basarab et al., 2011). Consequently, RFI has become an increasingly important parameter in the genetic improvement of feed efficiency in ruminants.

The rumen and hindgut are the two most important fermentation sites in ruminants (Russell and Hespell, 1981). The rumen, a digestive organ unique to ruminants, harbors a complex microbial community capable of degrading cellulose and synthesizing volatile fatty acids (VFAs), amino acids, and other essential nutrients to supply energy to the host (Tellez et al., 2006; Abecia et al., 2018; Zhang et al., 2018; Krautkramer et al., 2021). In contrast, the rectum, as the terminal section of the digestive tract, offers an integrated reflection of the metabolic effects of microorganisms and the host’s energy utilization efficiency (Du et al., 2023). Previous studies have demonstrated that gut microorganisms play critical roles in feed conversion efficiency, milk production, fat deposition, and growth performance in animals (Delgado et al., 2019; Noel et al., 2019; Clemmons et al., 2019). In recent years, metagenomic techniques have become powerful tools for elucidating the diversity and functional potential of gut microbiota (Shi et al., 2022). Untargeted metabolomics enables the systematic capture of dynamic changes in metabolites derived from both the host and microorganisms, facilitating the exploration of interactions between them (Meale et al., 2017). Integrated analyses combining metagenomics and metabolomics have increasingly been employed to investigate the relationship between the gut microecosystem and the host, offering new perspectives for understanding the impact of gut microbiota on host metabolism and production performance (Liu et al., 2022; Wang et al., 2021). At present, studies on feed efficiency in ruminants have predominantly focused on rumen microbiota, while systematic investigations into the coordinated regulatory mechanisms of the rumen and hindgut remain limited. Therefore, in this study, polytocous fine-wool sheep were grouped according to their RFI values, and both rumen and rectal contents were collected for metagenomic sequencing and LC–MS-based untargeted metabolomic analysis. The objectives were to systematically characterize the microbial and metabolic differences between intestinal segments, construct a “microbiota–metabolite–RFI phenotype” association network, and elucidate the molecular mechanisms underlying multi-segment coordination in the regulation of feed efficiency, thereby providing a theoretical basis for efficient breeding and precision nutritional regulation in polytocous fine-wool sheep.

## 2 Materials and methods

### 2.1 Experimental design and sample collection

All experimental animals were provided by Xingke Livestock Co., Ltd., Baicheng County, Aksu City, Xinjiang Uygur Autonomous Region, China. To control for the potential confounding effects of sex and associated physiological states (e.g., estrous cycle, pregnancy, and lactation) on growth performance, ruminal function, and metabolite profiles, we exclusively selected 70 clinically healthy male lambs from a polytocous fine-wool sheep population, all with detailed pedigree records and closely matched birth dates. Prior to the experimental feeding period, all lambs were immunized and dewormed according to the farm’s standard procedures. They were fed a uniform complete pelleted diet and had ad libitum access to water. The experiment consisted of three phases: a 14-day transition period, a 10-day pre-feeding period, and a 100-day formal experimental period, which was further divided into five 20-day stages. During the formal period, the daily feed intake of each lamb was precisely recorded, and body weight and body measurements were taken in the morning after an overnight fast on days 0, 20, 40, 60, 80, and 100 to evaluate growth performance. At the end of the experiment, lambs were slaughtered for sampling, and rumen and rectal contents (i.e., material collected directly from the rectal lumen) were immediately snap-frozen in liquid nitrogen and stored at −80 °C to preserve biological activity and prevent RNA degradation. At the end of the experiment, rumen and rectal contents were collected from all lambs, snap-frozen in liquid nitrogen, and stored at −80 °C to preserve biological activity and prevent RNA degradation. All sampling procedures strictly followed the guidelines for the care and use of animals to ensure animal welfare.

**Figure 1:**
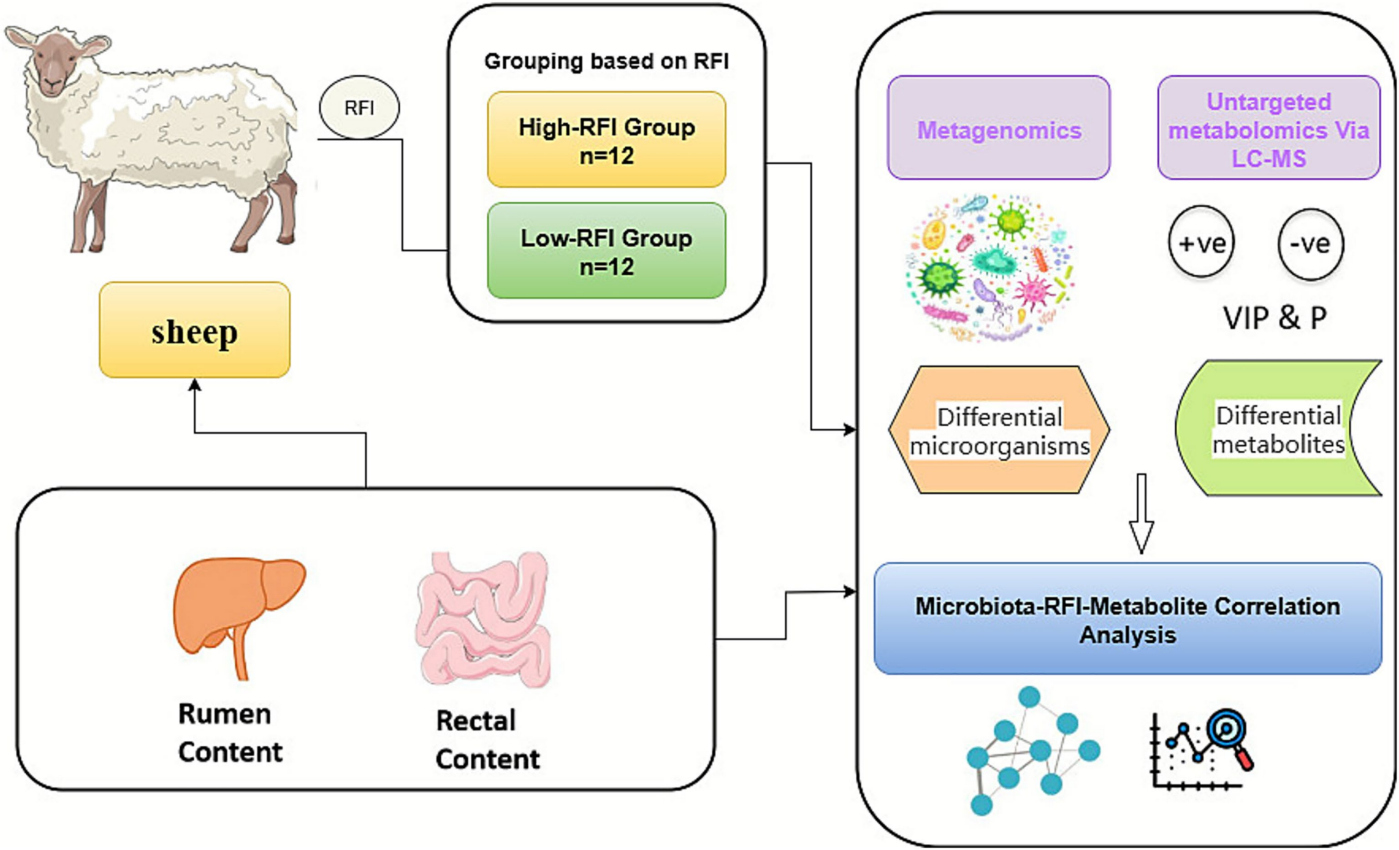
Experimental design and sample collection.

To further explore the relationship between feed efficiency and the characteristics of gut microbiota and metabolites, an extreme-phenotype grouping strategy based on residual feed intake (RFI) was employed. Lambs were ranked by residual feed intake (RFI) values from highest to lowest. The 12 lambs with the lowest RFI values were assigned to the low-RFI (LRFI) group, and the 12 with the highest values to the high-RFI (HRFI) group. In total, 24 lambs were used for subsequent integrated microbiome and metabolomics analyses. This extreme-phenotype strategy has been widely applied and validated in previous studies, and it provides a more effective means of accentuating the biological differences between high- and low-RFI individuals that are related to feed efficiency. Consequently, it enhances the detectability and statistical power for identifying key microbial and metabolic features in microbiome and metabolomics analyses. The experimental design and sample collection are shown in Figure 1.

### 2.2 Measurement of animal growth performance

Throughout the experiment, the initial body weight (IBW), final body weight (FBW), and daily dry matter intake (DMI) of each lamb were recorded. Growth performance traits, including average daily gain (ADG), average daily feed intake (ADFI), mid-test metabolic body weight (MBW), residual feed intake (RFI), and feed conversion ratio (FCR), were subsequently calculated. The RFI was determined using a multiple linear regression model in SPSS 26.0, as shown in the following equation:


$$\mathrm{MBW}={\left(\frac{{\mathrm{IBW}}_{i}+{\mathrm{FBW}}_{i}}{2}\right)}^{0.75}$$

$${\mathrm{ADG}}_{i}=\frac{{\mathrm{FBW}}_{i}-{\mathrm{IBW}}_{i}}{N}$$

$$Y_{i}=\beta_{0}+\beta_{1}({\mathrm{ADG}}_{i})+\beta_{2}{\mathrm{MBW}}_{i}+e_{i}$$

In the equation, MBW is the mid-test metabolic body weight of animal *i*; FBW, the final body weight of animal *i*; IBW, the initial body weight of animal *i*; *N*, the number of experimental days; and *Y*, the actual dry matter intake of animal *i*. *β*₀ is the regression intercept; *β*₁ is the regression coefficient for MBW; and *β*₂ is the regression coefficient for ADG. *e* represents the difference between the actual and predicted feed intake of animal *i*, which corresponds to the RFI value.

### 2.3 DNA isolation and library construction

Total DNA was isolated from sample using a QIAamp® Fast DNA Stool Mini Kit (Qiagen, Hilden, Germany) following the manufacturer’s instructions. DNA concentration and integrity were assessed by a NanoDrop2000 spectrophotometer (Thermo Fisher Scientific, Waltham, MA, United States) and agarose gel electrophoresis, respectively. DNA was fragmented by S220 Focused-ultrasonicators (Covaris, United States) and cleaned up by Agencourt AMPure XP beads (Beckman Coulter Co., United States). Then the libraries were constructed using TruSeq Nano DNA LT Sample Prepararion Kit (Illumina, San Diego, CA, United States) according to the manufacturer’ s instructions. The libraries were sequenced on an llumina Novaseq 6000 platform and 150 bp paired-end reads were generated The Metagenome sequencing was conducted by OE Biotech Co., Ltd. (Shanghai, China).

### 2.4 Bioinformatics analysis of metagenomic sequencing data

Raw sequencing data (FastQ files) were quality-controlled using fastp (v0.20.1) to remove low-quality bases and adapter sequences. The quality-filtered paired-end reads were then aligned to the sheep reference genome using Bowtie2 (v2.3.5.1) to remove host-derived sequences. Non-host reads were retained and assembled into contigs using MEGAHIT (v1.2.9). Scaffolds were broken at internal gaps to generate new contigs (Scaftigs), and those longer than 500 bp were kept for further analysis. Open reading frames (ORFs) were predicted from the assembled scaffolds using Prokka (v2.6.3) and translated into amino acid sequences. Non-redundant gene sets were constructed by clustering all predicted genes using CD-HIT (v4.8.1) at 95% sequence identity and 90% coverage thresholds, with the longest gene selected as the representative sequence of each cluster. Clean reads from each sample were mapped back to the non-redundant gene set using Salmon (v1.8.0) with a 95% identity threshold to quantify gene abundance, which was expressed as transcripts per million (TPM).

Functional annotation was performed using eggNOG-mapper (v2.1.9), with annotation databases including the Kyoto Encyclopedia of Genes and Genomes (KEGG) and the Carbohydrate-Active enZYmes (CAZy) database. Based on the functional annotation results, a functional abundance matrix was constructed. Statistical tests were then applied to identify significantly different functions between groups (*p* < 0.05). The differential results were visualized using STAMP software. Using Kraken2 software, species classification and annotation were performed. Taxonomic classification of sequences was performed using Kraken2. Abundance of taxa at multiple taxonomic levels—Domain, Kingdom, Phylum, Class, Order, Family, Genus, and Species—was quantified across samples to generate abundance profiles at each level. Alpha diversity indices were calculated using MOTHUR (v1.30.1). Principal coordinates analysis (PCoA) was performed on the taxonomic abundance profiles and visualized using R software (v4.1.2). Differential taxa analysis was conducted using Linear Discriminant Analysis Effect Size (LEfSe).

### 2.5 Untargeted metabolomics sequencing

For metabolite extraction, 60 mg of each sample was placed into a 1.5 mL centrifuge tube, followed by the addition of two small steel beads and 600 μL of methanol–water solution (4:1, v/v) containing mixed internal standards at a concentration of 4 μg/mL. Samples were pre-cooled at −40 °C for 2 min and then homogenized at 60 Hz for 2 min using a fully automated rapid sample grinder. Subsequently, ultrasonic extraction was performed in an ice-water bath for 10 min, and samples were left at −40 °C for 2 h to precipitate impurities. The mixture was centrifuged at 13,000 rpm and 4 °C for 20 min using a benchtop high-speed refrigerated centrifuge. A 150 μL aliquot of the supernatant was collected using a syringe, filtered through a 0.22 μm organic-phase syringe filter, and transferred into LC–MS vials for storage at −80 °C until analysis. Quality control (QC) samples were prepared by mixing equal volumes of all sample extracts to evaluate instrument stability and data quality. Untargeted metabolomics analysis was performed using a Waters ACQUITY UPLC I-Class PLUS coupled to a Thermo QE HF high-resolution mass spectrometer (UHPLC-HRMS), equipped with an ACQUITY UPLC HSS T3 column (100 mm × 2.1 mm, 1.8 μm). Chromatographic conditions were as follows: column temperature 45 °C, mobile phase A, water with 0.1% formic acid; mobile phase B, acetonitrile; flow rate 0.35 mL/min; injection volume 2 μL. In the metabolomics analysis, one rumen sample from the high-RFI group was damaged, resulting in 11 samples for this group, while the other groups each included 12 samples.

### 2.6 Bioinformatics analysis of untargeted metabolomics data

Raw metabolomics data were preprocessed using Progenesis QI v3.0 (Nonlinear Dynamics, Newcastle, UK), including baseline correction, peak detection, peak area extraction, retention time alignment, peak alignment, and normalization. For data cleaning, metabolic features with more than 50% missing values within a group were removed. Remaining missing values, including zeros, were imputed using half of the minimum non-zero value across all samples to minimize technical bias. The peak area data were subsequently subjected to total peak area normalization. In this process, the peak area of each metabolite was divided by the sum of all peak areas within the same sample to correct for overall response variability across samples. Following this, metabolite annotation and identification were performed using the HMDB, LipidMaps (v2.3), METLIN, and a custom-built local database, LuMet-Animal3.0. To ensure the reliability of metabolite annotation, identifications were performed following the Metabolomics Standards Initiative (MSI) guidelines, which classify annotation confidence into four levels (Level 1–4) primarily based on retention time deviation and MS/MS fragmentation pattern matching. Differential metabolites and subsequent biological analyses in this study primarily relied on high-confidence annotations (Levels 1–3), and low-confidence results were excluded based on a quality score threshold (Score ≥ 36/80), thereby ensuring the robustness of the metabolomic data. Downstream statistical analysis and functional enrichment were carried out on the MetaboAnalyst 5.0 platform (Pang et al., 2021), including metabolite selection, multivariate statistical analysis (PCA and OPLS-DA), and KEGG pathway enrichment analysis. Differential metabolites were selected based on a variable importance in projection (VIP) value > 1 in the OPLS-DA model and a *p*-value < 0.05 from t-tests. The selected differential metabolites were then used to reconstruct the OPLS-DA model for sample classification, and PCA plots were generated to evaluate clustering. Top 15 VIP-ranked metabolites were visualized using lollipop plots generated in R (v4.2.0) with the ggplot2 package to highlight key metabolites in the OPLS-DA model. KEGG pathway enrichment results were visualized as bubble plots using the clusterProfiler and ggplot2 packages, showing the degree of pathway enrichment and statistical significance.

### 2.7 Microbe–metabolite–RFI correlation analysis

To explore potential microbe–metabolite interactions associated with residual feed intake (RFI) across different gut niches, a hierarchical screening strategy was applied to construct interaction networks for the rumen and rectum, respectively: (1) Spearman correlations between differential microbial taxa identified by LEfSe (*p* < 0.05 and |LDA| > 2) and RFI were calculated for the rumen and rectum. Microbes with |*r*| > 0.5 and *p* < 0.05 in the rumen (4 taxa) and |*r*| > 0.6 and *p* < 0.05 in the rectum (16 taxa) were retained. (2) Core metabolites, including the top 15 differential metabolites ranked by VIP (VIP > 1 and *p* < 0.05) and metabolites from significantly enriched KEGG pathways (*p* < 0.05), were integrated. Spearman correlations with RFI were calculated, and metabolites significantly associated with RFI (|*r*| > 0.5, *p* < 0.05) were selected, resulting in 15 and 23 target metabolites for the rumen and rectum, respectively. (3) Spearman rank correlations between the selected microbial and metabolite sets were performed for each site using the R package psych (v2.3.6). Results were visualized as heatmaps (pheatmap v1.0.12) and interaction networks (igraph v1.5.1), with orange and green edges representing positive and negative correlations, respectively.

## 3 Results

### 3.1 Growth performance of polytocous fine-wool lambs with different RFI

The growth performance of polytocous fine-wool lambs is presented in Table 1. The HRFI and LRFI groups did not differ significantly in initial body weight (IBW), final body weight (FBW), mid-term metabolic body weight (MBW), or average daily gain (ADG) (*p* > 0.05). In contrast, average daily feed intake (ADFI), residual feed intake (RFI), and feed conversion ratio (FCR) differed highly significantly between the groups (*p* < 0.001), with the HRFI group showing significantly higher ADFI and RFI than the LRFI group.

**Table 1:** Growth performance of polytocous fine-wool lambs.

Item	High RFI	Low RFI	SEM	*P-*value
IBW (kg)	19.48	18.38	1.06	0.31
FBW (kg)	45.32	43.64	1.72	0.34
MBW (kg)	13.57	13.12	0.41	0.29
ADG (kg/d)	0.29	0.28	0.01	0.64
ADFI (kg/d)	1.64	1.44	0.04	1.29E-04
RFI (kg/d)	0.09	−0.07	0.01	2.23E-10
FCR	5.63	5.09	0.18	7.89E-03

IBW, initial body weight; FBW, final body weight; MBW, mid-test body weight; ADG, average daily gain; ADFI, average daily feed intake; RFI, residual feed intake; FCR, feed conversion ratio; SEM, standard error of the mean; *P* values were obtained by independent-samples *t*-test. *P* < 0.05 indicates statistically significant differences between groups.

### 3.2 Microbial diversity

**Figure 2:**
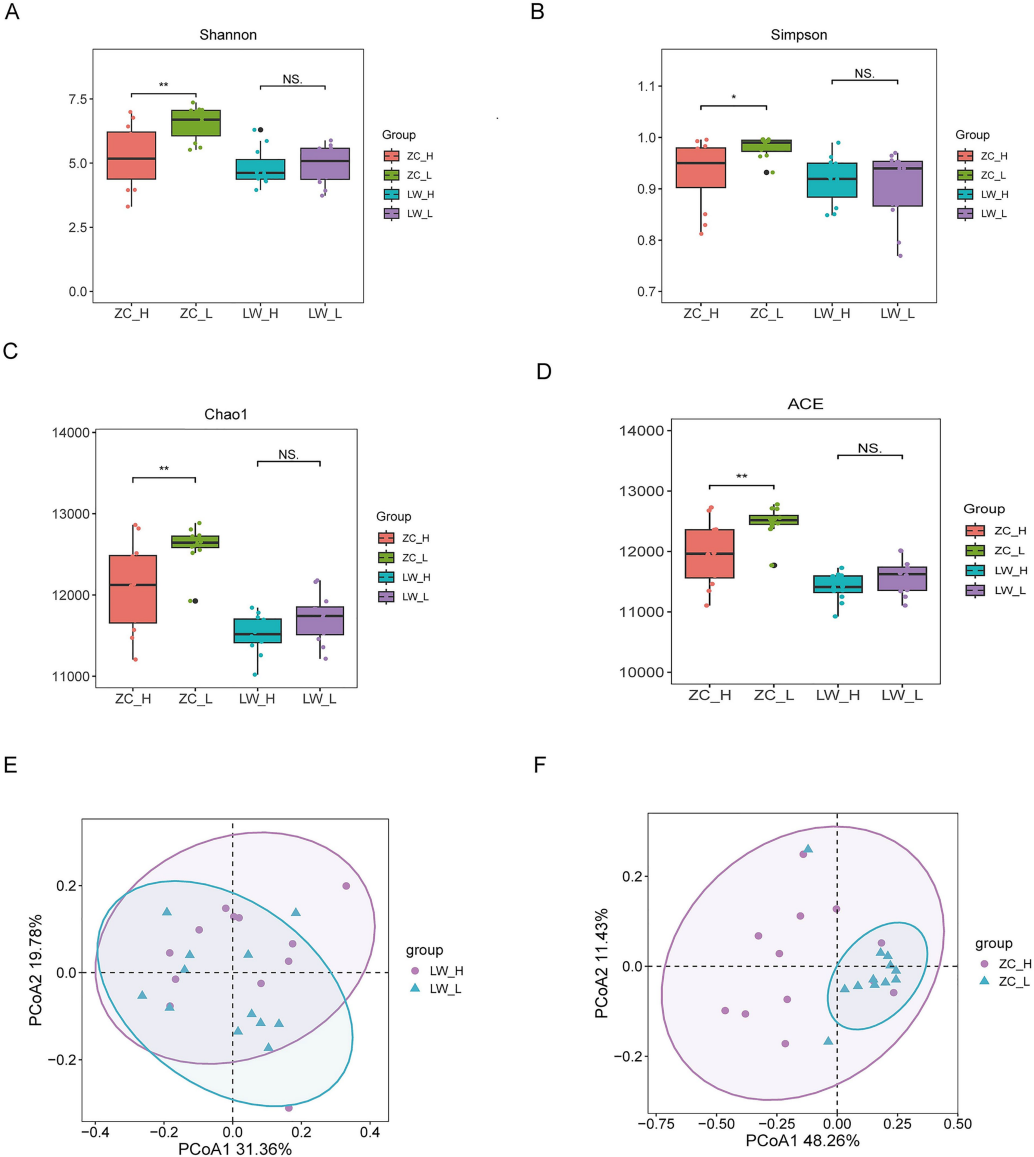
Microbial diversity. **(A)** Shannon index; **(B)** Simpson index; **(C)** Chao1 index; **(D)** ACE index; **(E)** PCoA analysis of rumen microorganisms; **(F)** PCoA analysis of rectal microorganisms.

Rumen and rectum samples (*n* = 24 per tissue, 12 high-RFI and 12 low-RFI) were subjected to metagenomic sequencing. For rumen samples, 1,920,903,634 raw reads were obtained, and 1,920,386,402 clean reads were retained after quality control, with an average of approximately 80,016,100 reads per sample. Assembly generated 4,219,046 contigs, with an N50 of 102,703 bp and an average GC content of 46.82% (Supplementary Table S1). For rectum samples, 1,909,559,074 raw reads were obtained, and 1,909,045,306 clean reads were retained after quality control, with an average of approximately 79,543,554 reads per sample. Assembly produced 8,686,554 contigs, with an N50 of 61,280 bp and an average GC content of 42.23% (Supplementary Table S2). These results indicate that the sequencing and assembly were of high quality. Alpha diversity analysis of the microbial communities showed no significant differences in Chao1, Shannon, Simpson, or ACE indices between the high-RFI (HRFI) and low-RFI (LRFI) groups in the rumen (*p* > 0.05) (Figures 2A–D). In contrast, the rectal microbial communities exhibited significant differences in alpha diversity between RFI groups (*p* < 0.05), with the LRFI group showing higher species richness and diversity. Principal coordinate analysis (PCoA) based on Bray–Curtis distances revealed partial overlap of microbial communities in the rumen and rectum among lambs with different RFI values, with no distinct separation, suggesting only minor differences in microbial composition between groups (Figures 2E,F).

### 3.3 Taxonomic composition analysis

**Figure 3:**
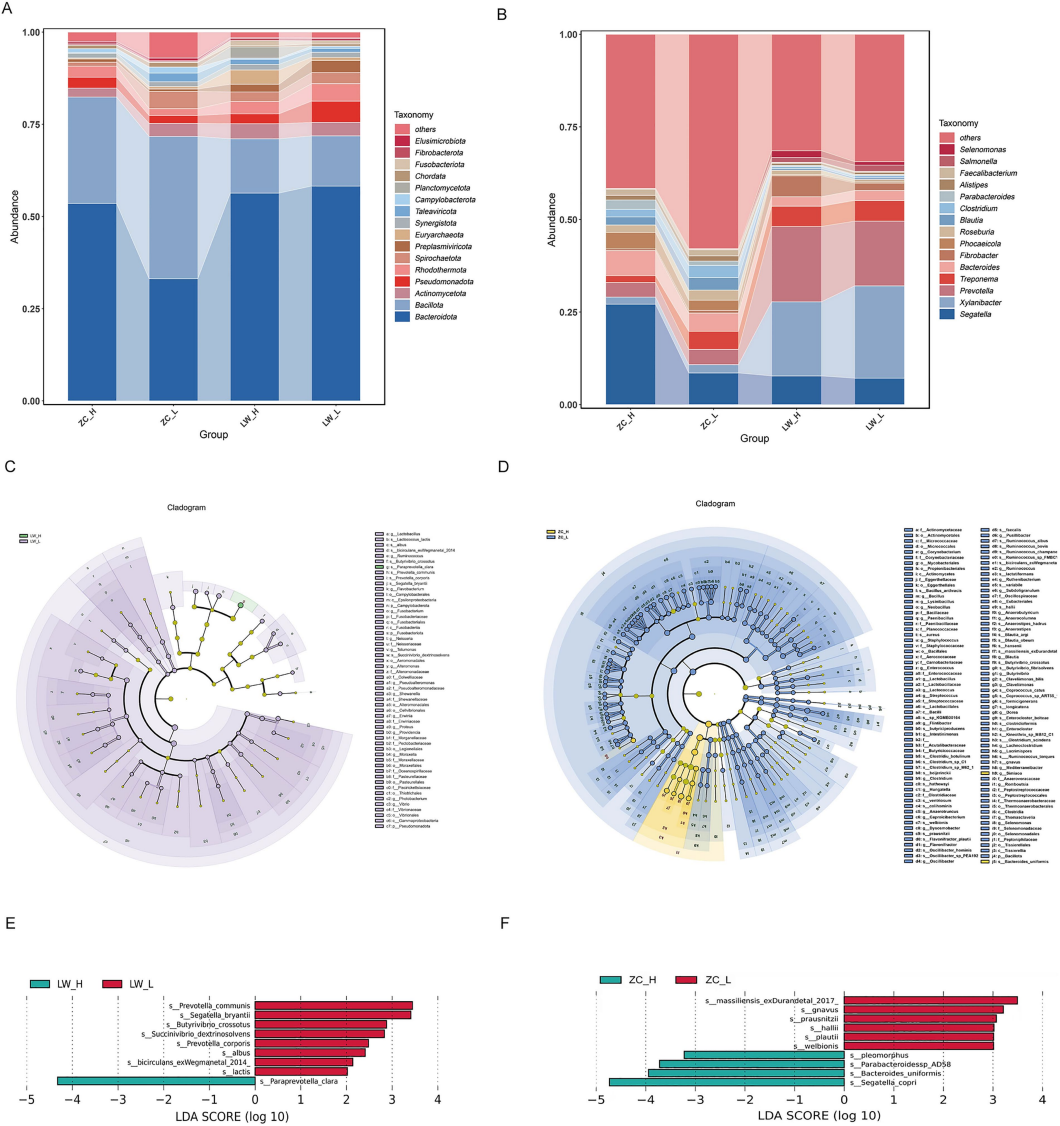
Taxonomic composition. **(A)** Relative abundance of the top 15 dominant phyla in rumen and rectal samples. **(B)** Relative abundance of the top 15 dominant genera in rumen and rectal samples. **(C)** LEfSe cladogram of the rumen microbiota. **(D)** LEfSe cladogram of the rectal microbiota. **(E)** LEfSe analysis of rumen microbiota showing significantly discriminative microbial biomarkers between RFI groups (LDA score > 2.0, *p* < 0.05). **(F)** LEfSe analysis of rectal microbiota showing significantly discriminative microbial biomarkers between RFI groups (LDA score > 3.0, *p* < 0.05).

Analysis of rumen and rectal microbial composition revealed that, at the phylum level, dominant taxa in both the rumen and rectum were similar, primarily comprising Bacteroidota, Firmicutes, and Actinobacteriota, which together accounted for over 70% of the total relative abundance. In the rumen, Bacteroidota and Firmicutes accounted for 58.2 and 13.5% in the LRFI group and 56.3 and 14.7% in the HRFI group, respectively, with Chordata significantly higher in the HRFI group than in the LRFI group (*p* < 0.05), while no significant differences were observed for other phyla (*p* > 0.05). In the rectum, Bacteroidota and Firmicutes represented 33.1 and 38.5% in the LRFI group and 53.5 and 28.8% in the HRFI group, respectively, with Bacteroidota significantly enriched in the HRFI group (*p* < 0.05) (Figure 3A; Supplementary Table S3). At the genus level, Segatella, Xylanibacter, and Prevotella were the predominant taxa in both rumen and hindgut. Whereas other genera exhibited no significant differences (*p* > 0.05). In the rectum, among the top 15 genera, Parabacteroides, Phocaeicola, Segatella, and Bacteroides were significantly more abundant in the HRFI group compared with the LRFI group (*p* < 0.05) (Figure 3B; Supplementary Table S4). Differential microbial taxa between RFI groups were identified using the LEfSe method with thresholds of LDA ≥ 2.0 and *p* < 0.05. In the rumen, 54 differential taxa were identified (Figure 3C; Supplementary Table S5), including 9 species-level taxa (Figure 3E). In the rectum, 182 differential taxa were detected (Figure 3D; Supplementary Table S6), of which 49 were species-level, including 10 taxa with LDA ≥ 3.0 and *p* < 0.05, indicating their strong discriminative power between groups (Figure 3F).

### 3.4 Microbial functional annotation

**Figure 4:**
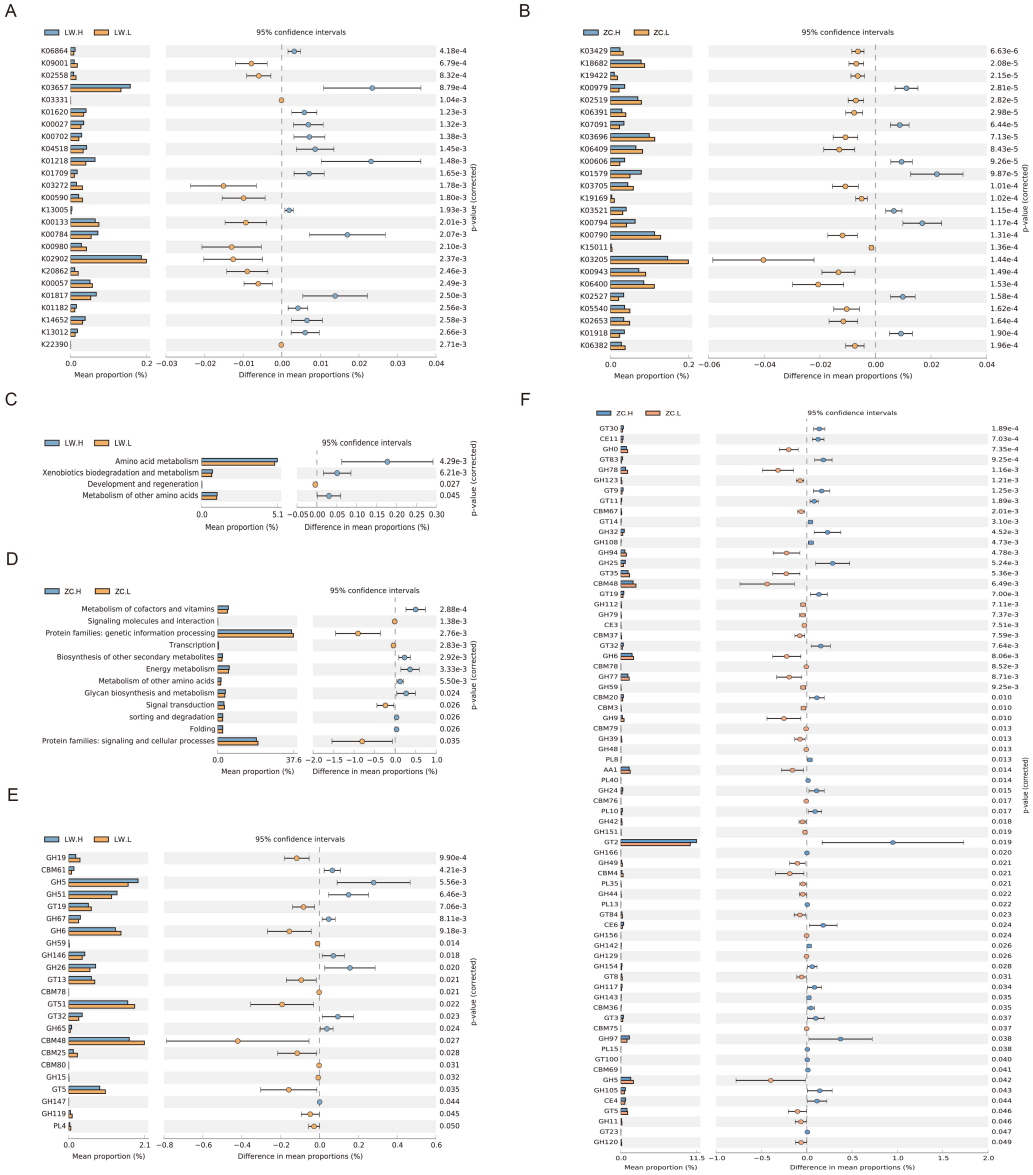
Microbial functional annotation. **(A)** Top 25 differential KEGG Orthology (KO) annotations in the rumen between different RFI groups. **(B)** Top 25 differential KEGG Orthology (KO) annotations in the rectum between different RFI groups. **(C)** Differences in functional abundance at the KEGG secondary pathway level in the rumen. **(D)** Differences in functional abundance at the KEGG secondary pathway level in the rectum. **(E)** Differences in gene abundance at the CAZy secondary classification level in the rumen. **(F)** Differences in gene abundance at the CAZy secondary classification level in the rectum. All results were visualized using extended error bar plots generated by STAMP software, with error bars representing 95% confidence intervals. Intergroup differences were assessed using Welch’s *t*-test (*p* < 0.05).

To investigate microbial functions associated with feed efficiency in polytocous fine-wool sheep, gene functional annotation was performed based on the KEGG and CAZy databases. A total of 7,825 and 8,815 KEGG genes were identified in the rumen and rectum, respectively. Among them, 602 differentially homologous genes were detected in the rumen (*p* < 0.05), and 1,569 differentially homologous genes were identified in the rectum (*p* < 0.05). The top 25 differential KOs with the most significant *p*-values were visualized for each tissue. In the rumen, key differential KOs enriched in the low residual feed intake group (LRFI, high feed efficiency) were mainly involved in glycan biosynthesis and metabolism (K03272), signaling and cellular processes (K00590), and lipid metabolism (K00057, K00980), whereas KOs enriched in the high residual feed intake group (HRFI, low feed efficiency) were primarily associated with replication and repair (K03657), carbohydrate metabolism (K00027, K00702, K01218, K01182, K01709), and protein families metabolism (K13005) (Figure 4A; Supplementary Table S7). In the rectum, LRFI-enriched differential KOs were related to genetic information processing (K03696, K03705, K05540, K02519), signaling and cellular processes (K06409, K02653, K19169), and signal transduction (K15011), while HRFI-enriched KOs were associated with metabolism of cofactors and vitamins (K01579, K01918, K00794) and glycan biosynthesis and metabolism (K00979, K02527) (Figure 4B; Supplementary Table S8).

KEGG pathway analysis further indicated that in the rumen, pathways related to other amino acid metabolism and xenobiotics biodegradation and metabolism were significantly enriched in the HRFI group (*p* < 0.05), whereas pathways associated with development and regeneration were significantly enriched in the LRFI group (*p* < 0.05) (Figure 4C). In the rectum, HRFI-enriched pathways included protein folding, sorting, and degradation, carbohydrate synthesis and metabolism, energy metabolism, and cofactor and vitamin metabolism (*p* < 0.05), while pathways associated with signaling molecules and interactions, genetic information processing, transcriptional regulation, and signal transduction were significantly enriched in the LRFI group (*p* < 0.05) (Figure 4D). CAZy-based analysis of carbohydrate-active enzyme (CAZyme) genes revealed 253 and 273 CAZyme-related genes in the rumen and rectum microbiota, respectively. In the rumen, 23 differential genes exhibited significant differences between RFI groups (*p* < 0.05), with 14 enriched in the LRFI group and 9 in the HRFI group (Figure 4E). In the rectum, 70 differential genes showed significant intergroup differences (*p* < 0.05), including 37 enriched in the LRFI group and 33 in the HRFI group. Across both tissues, the GH and GT family genes were the most abundant, and the number of differential genes in the LRFI group exceeded that in the HRFI group (Figure 4F).

### 3.5 Differential metabolite analysis

**Figure 5:**
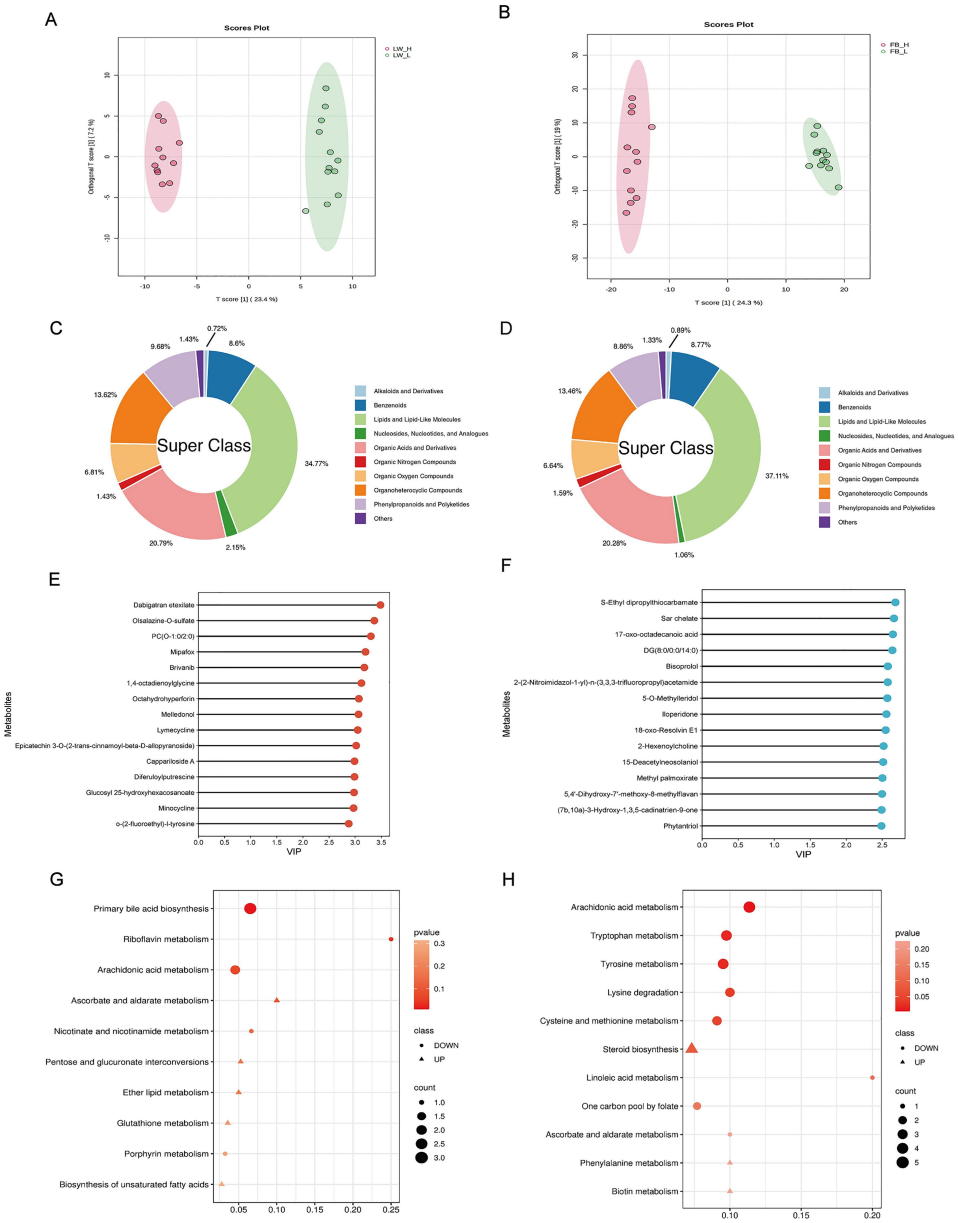
Differential metabolite features in the rumen and rectum samples. **(A)** OPLS-DA score plot of rumen. **(B)** OPLS-DA score plot of rectum samples. **(C)** Proportions of significantly differential metabolites in the rumen. **(D)** Proportions of significantly differential metabolites in the rectum. **(E)** Top 15 differential metabolites in the rumen ranked by VI*p* values. **(F)** Top 15 differential metabolites in the rectum ranked by VIP values. **(G)** KEGG pathway enrichment analysis of differential metabolites in the rumen. **(H)** KEGG pathway enrichment analysis of differential metabolites in the rectum.

Metabolites in the rumen and rectum of polytocous fine-wool sheep with different RFI levels were identified by LC–MS sequencing. In the rumen, 279 differential metabolites were detected (VIP > 1 and *p* < 0.05), including 113 upregulated and 166 downregulated metabolites (Supplementary Table S9). In the rectum, 1,129 differential metabolites were identified (VIP > 1 and *p* < 0.05), of which 528 were upregulated and 601 downregulated (Supplementary Table S10). Category analysis of differential metabolites (Figures 5C,D) showed that lipids and lipid-like molecules accounted for the largest proportion in both the rumen and rectum (34.8 and 37.1%, respectively), followed by organic acids and derivatives (20.8 and 20.3%) and organic heterocyclic compounds (13.6 and 13.5%). To evaluate the discriminative capability of the selected differential metabolites, OPLS-DA models were constructed separately for the rumen and rectum based on these metabolites. The resulting score plots revealed a clear separation between the high- and low-RFI groups, indicating that these differential metabolites possess strong discriminative power (Figures 5A,B). The OPLS-DA model for the rumen showed R^2^Y = 0.935 and Q^2^ = 0.897, whereas the rectum model showed R^2^Y = 0.645 and Q^2^ = 0.576. Following 1,000 permutation tests (*p* < 0.001), the results confirmed that both models were robust and free from overfitting (Supplementary Figure S1). The top 15 differential metabolites ranked by VIP values in the rumen and rectum were selected for subsequent correlation analysis with differential microbiota (Figures 5E,F). KEGG pathway enrichment analysis, performed using the Pathway Analysis module in MetaboAnalyst, revealed that only two pathways were significantly enriched in the rumen (*p* < 0.05)—primary bile acid biosynthesis and riboflavin metabolism—both showing downregulation in the LRFI group (Figure 5G). In the rectum, five pathways were significantly enriched (*p* < 0.05), including arachidonic acid metabolism, tryptophan metabolism, tyrosine metabolism, lysine degradation, and cysteine and methionine metabolism, all exhibiting downregulation in the LRFI group (Figure 5H).

### 3.6 Correlation analysis between differential microbiota, RFI, and differential metabolites

**Figure 6:**
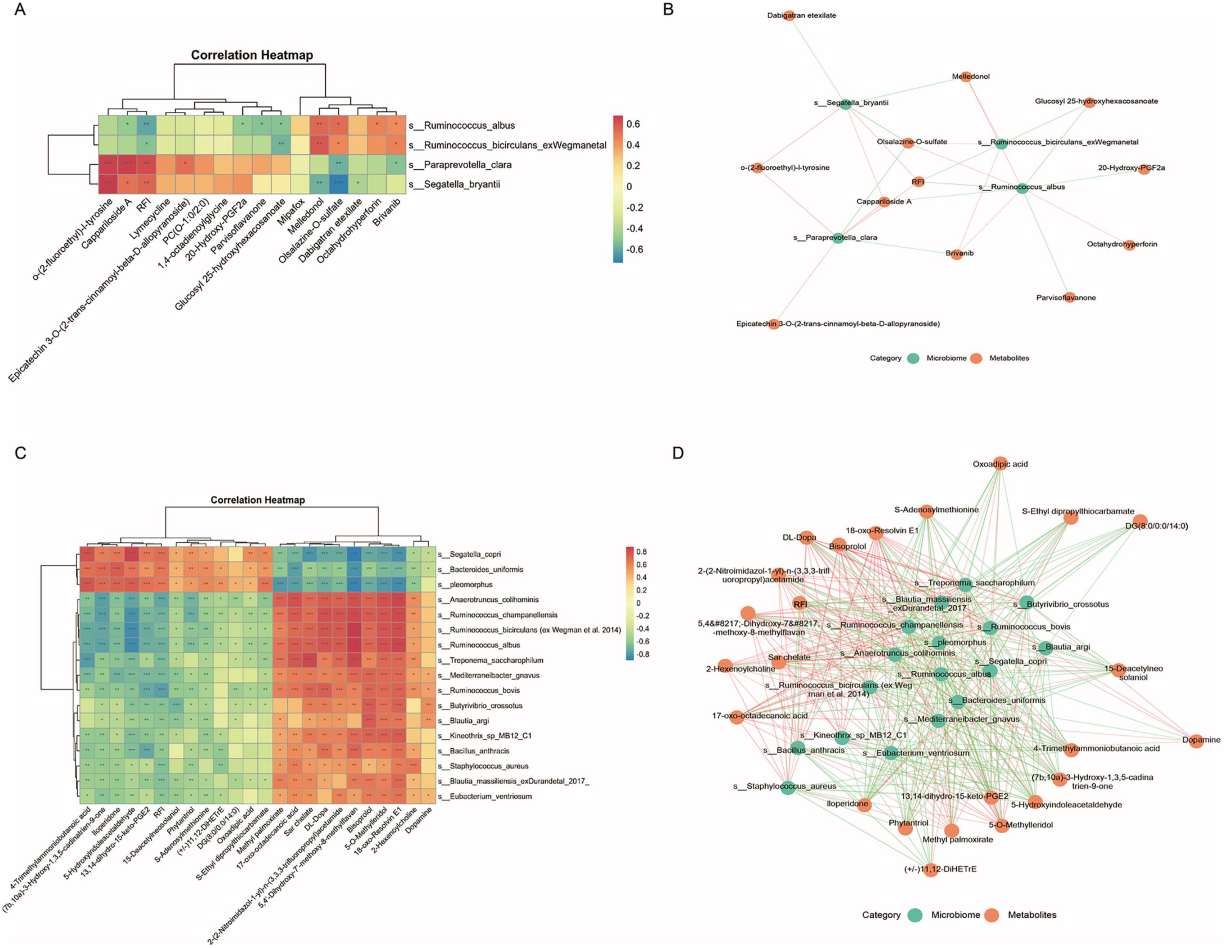
Correlation analysis between differential microbiota, RFI, and differential metabolites. **(A)** Heatmaps showing correlations between differential microbes and differential metabolites in the rumen. **(B)** Interaction networks of differential microbes and differential metabolites in the rumen. **(C)** Heatmaps showing correlations between differential microbes and differential metabolites in the rectum. **(D)** Interaction networks of differential microbes and differential metabolites in the rectum.

Based on correlation analysis, several key microbes significantly associated with RFI were selected to further explore their relationships with specific metabolites, aiming to reveal potential regulatory mechanisms. In the rumen, *s_Paraprevotella_clara* was significantly positively correlated with RFI and also positively correlated with Epicatechin 3-O-(2-trans-cinnamoyl-β-D-allopyranoside), o-(2-fluoroethyl)-L-tyrosine, and Cappariloside A, but negatively correlated with Olsalazine-O-sulfate and Brivanib. *s_Ruminococcus_albus* was significantly negatively correlated with RFI, positively correlated with Octahydrohyperforin, Melledonol, Olsalazine-O-sulfate, and Brivanib, and negatively correlated with 20-Hydroxy-PGF2a, Parvisoflavanone, Glucosyl 25-hydroxyhexacosanoate, and Cappariloside A (Figure 6A). To investigate the associations between key microbes and metabolites in the rumen, a microbe–metabolite interaction network was constructed based on significant correlations. The network comprised 4 microbial nodes and 16 metabolite nodes, including RFI, connected by 26 significant edges.*s_Ruminococcus_albus* and *s_Ruminococcus_bicirculans*, which were negatively correlated with RFI, and *s_Segatella_bryantii* and *s_Paraprevotella_clara*, which were positively correlated with RFI, exhibited multiple significant associations with differential metabolites. Notably, *s_Ruminococcus_albus* functioned as a hub node, establishing extensive linkages with 9 differential metabolites and constituting a highly connected core interaction module within the rumen network (Figure 6B).

In the rectum, *s_Anaerotruncus colihominis, s_Ruminococcus albus*, and *s_Ruminococcus_bicirculans* were significantly negatively correlated with RFI and positively correlated with 18-oxo-Resolvin E1, 17-oxo-octadecanoic acid,5,4’-Dihydroxy-7′-methoxy-8-methylflavan, and 5-O-Methylleridol, but negatively correlated with (7b,10a)-3 -Hydroxy-1,3,5-cadinatrien-9-one, 5-Hydroxyindoleacetaldehyde, 4-Trimethy- lammoniobutanoic acid, and 13,14-dihydro-15-keto-PGE2. Conversely, *s_Bacteroides_uniformis* and *s_Segatella_copri* were positively correlated with RFI and also positively correlated with Iloperidone, (7b,10a)-3-Hydroxy-1,3,5-cadinatrien-9-one, 5-Hydroxyindoleacetaldehyde, and 4-Trimethylammoniobutanoic acid, while negatively correlated with 5,4’-Dihydroxy-7′-methoxy-8-methylflavan, 17-oxo-octadecanoic acid, 18-oxo-Resolvin E1, and 5-O-Methylleridol (Figure 6C). To further elucidate the associations between key rectal microbes and metabolites, a differential microbe–metabolite interaction network was constructed based on statistically significant correlation coefficients. The network encompassed 17 microbial nodes and 24 metabolite nodes, including RFI, interconnected by 356 significant edges. Of the microbial nodes, three exhibited positive correlations with RFI, while the remaining 14 displayed negative correlations. *s_Ruminococcus_albus, s_Ruminococcus_bicirculans, s_Ruminococcus_champanellensis*, and *s_Anaerotruncus_colihominis* functioned as hub nodes, establishing extensive linkages with diverse lipid and polyphenol metabolites, thereby underscoring their central roles within the rectal network (Figure 6D).

## 4 Discussion

In recent years, with continuous advances in husbandry practices and improvements in environmental conditions, animal feed efficiency has been significantly enhanced. Feed efficiency is a key indicator for evaluating feed utilization capacity and the economic performance of livestock (Lin et al., 2023). In this study, we analyzed phenotypic differences between high- and low-RFI polytocous fine-wool sheep. The results showed that the low-RFI group exhibited significantly lower feed intake and higher feed conversion efficiency compared to the high-RFI group, indicating superior feed utilization. These findings are consistent with previous reports (Zhang et al., 2021; Metzler-Zebeli et al., 2017; Bai et al., 2022). Although it should be noted that RFI is a complex trait whose variation is shaped not only by husbandry conditions and gut microbial composition but also by the host’s genetic background. Previous studies have reported a heritability of 0.28–0.58 for RFI, indicating a moderate level of genetic control. Moreover, host genotypes can modulate intestinal immune homeostasis (Yang et al., 2025), barrier integrity, and metabolic signaling pathways (Dey, 2025), thereby directly or indirectly shaping the gut microbial community and jointly influencing the RFI phenotype. While the present study primarily focuses on identifying RFI-associated microbial taxa and metabolite alterations, and does not incorporate host-genetic analyses, the key microorganisms and metabolites identified here provide a valuable foundation for future integrative studies. Such efforts—combining metagenomics, metabolomics, and host genomic or transcriptomic data—will be essential for systematically elucidating how host genetic factors interact with the gut microbiome to affect RFI.

Metagenomics, as a core approach for profiling the gut microbiome, provides a critical perspective for elucidating host–microbe interactions (Shi et al., 2022). In this study, our results revealed a pronounced spatial heterogeneity in the gut microbial response to RFI in sheep. In the rumen, microbial α-diversity indices did not differ significantly between high- and low-RFI groups (*p* > 0.05). In contrast, rectal microbial diversity showed significant differences between the two RFI groups, with the LRFI group exhibiting markedly higher diversity than the HRFI group (*p* < 0.05), indicating a richer and more diverse microbial community in the LRFI rectum. These findings are consistent with previous studies (Ma et al., 2019). At the phylum level, Bacteroidetes and Firmicutes were the dominant phyla in both the rumen and rectum (Lv et al., 2021). Bacteroidetes and Firmicutes, as key functional phyla in the ruminant digestive tract, play essential roles in plant polysaccharide degradation, short-chain fatty acid (SCFA) production, nutrient transformation, and maintenance of intestinal homeostasis (Chen et al., 2011; Milligan et al., 1967; Zhang et al., 2021). Notably, in the rumen, the abundances of these three dominant phyla were higher in the LRFI group than in the HRFI group, whereas in the rectum, the opposite trend was observed. This gut-segment-specific differentiation in microbial structure suggests a site-dependent regulatory mechanism (Wang et al., 2024; Zhou et al., 2023). At the genus level, Segatella, Bacteroides, and Prevotella were the predominant genera in both the rumen and rectum. Prevotella can utilize other fiber-degrading microbes to break down cellulose into acetate and small amounts of isobutyrate, isovalerate, and lactate, thereby providing energy to the host (Ramayo-Caldas et al., 2018). In the rumen, the LRFI group showed higher abundances of Bacteroides and Prevotella, whereas Segatella was more abundant in the HRFI group. In the rectum, all three dominant genera were more abundant in the HRFI group. Therefore, the results indicate that the diversity and community structure of the gut microbiota differ markedly between RFI groups across intestinal segments. The LRFI group was enriched with microbial communities possessing strong potential for polysaccharide degradation and short-chain fatty acid (SCFA) production, suggesting enhanced carbohydrate utilization and energy acquisition, which may synergistically improve feed conversion efficiency by optimizing gut homeostasis. In contrast, microbial communities with polysaccharide-degrading and SCFA-producing potential were more abundant in the rectum of the HRFI group, possibly reflecting increased substrate load in the distal gut and the induction of a pro-inflammatory environment, thereby reducing energy utilization efficiency.

Metagenomic functional annotation reveals the metabolic network of microbial communities, providing a theoretical basis for elucidating the mechanisms by which microbes regulate host feed efficiency (Xue et al., 2022). Functional annotation results revealed that, in the rumen, the HRFI group showed significant enrichment in pathways related to other amino acid metabolism, xenobiotic biodegradation, and general amino acid metabolism, whereas the LRFI group was enriched in pathways associated with growth and development. In the rectum, the HRFI group was enriched in pathways related to protein folding, carbohydrate and amino acid metabolism, and energy metabolism, while the LRFI group showed enrichment in pathways involved in signaling molecules and interactions, transcriptional regulation, and signal transduction. These observations are consistent with findings in chicken cecal microbiota reported by Du et al. (2020), suggesting that efficient individuals may achieve higher resource utilization by enhancing local homeostatic regulation and signal integration. CAZyme functional annotation further revealed differences in carbohydrate metabolic potential. A total of 23 and 70 significantly differential CAZyme genes were identified in the rumen and rectum, respectively, with the LRFI group possessing more differential CAZyme genes than the HRFI group. This indicates a potentially stronger capacity for polysaccharide degradation and utilization in low-RFI individuals. A study reported that an increased abundance of CAZy enzymes (CAZymes) is closely associated with improved digestive capacity in pigs (Ramayo-Caldas et al., 2016). Notably, the GH (glycoside hydrolase) and GT (glycosyltransferase) families dominated the differential enzymes, participating in the degradation of polysaccharides such as cellulose and the reassembly of oligosaccharide structures, serving as key indicators of carbohydrate metabolic efficiency (Stewart et al., 2018; Su et al., 2022). Taken together, these results suggest that low-RFI individuals may enhance nutrient utilization from feed through superior microbial metabolic functions and fiber degradation potential, thereby promoting growth and improving feed conversion efficiency. Despite the KEGG and CAZy annotations revealing differences in microbial metabolic potential between sheep with divergent RFI values, these functional predictions currently lack empirical support at both the transcriptional and biochemical levels. To enhance the reliability of these inferred functions, future studies should incorporate multi-layered and complementary orthogonal validation strategies. For example, metatranscriptomic sequencing could be employed to assess the *in vivo* expression of key metabolic pathway genes, thereby determining whether the predicted functions are truly activated under physiological conditions. In addition, qPCR targeting representative CAZyme genes or key genes involved in short-chain fatty acid synthesis would enable verification of whether their expression patterns across RFI groups align with metagenomic predictions. Furthermore, direct measurement of enzyme activities in ruminal or rectal contents would provide the most immediate biochemical evidence for microbial metabolic functions. By integrating convergent evidence from gene expression profiling, targeted functional validation, and biochemical assays, future research will be able to more accurately and robustly elucidate the mechanistic links between microbial metabolic capacity and host feed efficiency.

Metabolomics enables the detection and quantification of small-molecule metabolites in cells, bodily fluids, and tissues, accurately reflecting dynamic changes in an organism’s metabolic state (Foroutan et al., 2019). In this study, lipids and lipid-like molecules dominated the metabolite profiles in both the rumen and rectum. As the primary energy source for sheep, fluctuations in these metabolites can markedly influence feed utilization efficiency (Kerr et al., 2015). In the rumen, the primary bile acid biosynthesis pathway was active. Bile acids and their derivatives play crucial roles in lipid emulsification and absorption, as well as in the regulation of energy metabolism and intestinal homeostasis (Zhou et al., 2024). Regarding fatty acid metabolism, α-linolenic acid (ALA), a precursor of *ω*-3 polyunsaturated fatty acids, was significantly elevated in the LRFI group, potentially further converted into long-chain polyunsaturated fatty acids such as eicosapentaenoic acid (EPA) and docosahexaenoic acid (DHA), which possess anti-inflammatory and lipid-regulatory functions (Calder, 2015). Metabolic features of rectal contents directly reflect the impact of the terminal gut microenvironment on host efficiency. The arachidonic acid metabolism pathway was significantly enriched, involving key metabolites such as 13,14-dihydro-15-keto-PGE2 and 5S-HETE. Arachidonic acid serves as a critical precursor of inflammatory mediators, with its metabolites, particularly prostaglandins, playing central roles in gut inflammatory responses (Korbecki et al., 2014; Guo et al., 2025). In this study, the pro-inflammatory mediator 13,14-dihydro-15-keto-PGE2 was elevated in the HRFI group, indicating increased local rectal inflammation. Gut inflammation can impair nutrient absorption and reduce feed conversion efficiency by reallocating energy and compromising barrier integrity. Another key pathway was tryptophan metabolism. Its metabolite 5-hydroxyindoleacetaldehyde serves as a precursor to serotonin (5-HT), a gut regulatory factor that modulates intestinal motility, immune homeostasis, and nutrient absorption (Wang et al., 2024; Yano et al., 2015). Collectively, these results indicate that optimized energy supply in the rumen, coupled with rectal anti-inflammatory and neuroregulatory mechanisms, constitutes a cross-gut metabolic hub driving differences in feed efficiency.

Phenotypic traits of animals are, to some extent, regulated by the gut microbiota (Zhang et al., 2017). In the present study, Pearson correlation analysis between differential microbes and metabolites was performed to explore the potential mechanisms by which dominant taxa influence feed efficiency. In the LRFI group, *s_Ruminococcus_albus*, a core fiber-degrading bacterium, was significantly enriched in both the rumen and rectum, and was negatively correlated with RFI. This species degrades plant cell wall polysaccharides efficiently by secreting cellulases and xylanases, thereby promoting acetate production and enhancing host energy utilization efficiency (Flint et al., 2008; Myer et al., 2015). Moreover, *s_Ruminococcus_albus* was positively correlated with the metabolites oxoadipic acid and DL-Dopa, suggesting that it may indirectly contribute to host energy absorption and amino acid metabolism through the promotion of key metabolites. Similarly, *s_Ruminococcus_champanellensis* and *s_Eubacterium_ventriosum* in the rectum were significantly enriched in the LRFI group. *s_Ruminococcus_champanellensis* possesses the potential to degrade dietary fibers and promote SCFA production (Cann et al., 2016), whereas *s_Eubacterium_ventriosum* is a typical butyrate-producing bacterium involved in butyrate synthesis, contributing to intestinal barrier integrity and energy homeostasis (Singh et al., 2023). Both taxa were positively correlated with anti-inflammatory metabolites (e.g., 18-oxo-Resolvin E1) and negatively correlated with proinflammatory metabolites (e.g., 13,14-dihydro-15-keto-PGE2), suggesting that they may enhance intestinal homeostasis and anti-inflammatory status by modulating related metabolites, thereby facilitating energy utilization (Yano et al., 2015). In contrast, *s_Paraprevotella_clara* and *s_Bacteroides_uniformis* were significantly enriched in the rumen and rectum of the HRFI group, respectively, and both were positively correlated with RFI. Previous studies have reported that *s_Paraprevotella_clara* modulates protease activity and bile acid metabolism, contributing to intestinal immune homeostasis (Li et al., 2022), whereas *s_Bacteroides_uniformis* is capable of degrading β-glucans, promoting the growth of lactic acid bacteria, and elevating indole-3-lactic acid levels to alleviate colonic inflammation in mice (Liu et al., 2024). In this study, these two species were enriched in the HRFI group and positively associated with pro-inflammatory metabolites, suggesting that the intestinal environment of HRFI animals may experience increased substrate load and a potentially mild pro-inflammatory state. The enrichment of anti-inflammatory bacteria may reflect a compensatory response of the gut microbiota, which in turn affects energy utilization efficiency. Collectively, the dominant taxa in the LRFI group appear to enhance feed conversion efficiency by promoting fiber degradation and SCFA production, as well as by reinforcing intestinal anti-inflammatory and homeostatic functions. These findings further suggest that targeted modulation of the gut microbial community may represent a promising strategy to improve production performance in ruminants.

Further correlation network analyses revealed not only robust associations between core microbial taxa and multiple metabolites in the low-RFI phenotype, but also highlighted a key ecological pattern. Specifically, we observed that key molecules, such as the anti-inflammatory metabolite 18-oxo-Resolvin E1, were significantly correlated with multiple distinct bacterial species (e.g., *s_Ruminococcus_albus*, *s_Ruminococcus_champanellensis*, and *s_Eubacterium_ventriosum*). This “one-to-many” correlation topology is a hallmark of functional redundancy in microbial communities, indicating that crucial metabolic functions are distributed across a consortium of species rather than being dependent on a single taxon. Moreover, this structure suggests the potential for compensatory mechanisms within the community: should the abundance of one functional species fluctuate, other species with similar functional capacities may compensate to maintain metabolic homeostasis. However, it is crucial to note that correlation analysis inherently identifies statistical co-occurrence and cannot establish causation. Therefore, to dissect the causal relationships along the microbiota-metabolite-feed efficiency axis and to validate the compensatory hypothesis proposed above, future studies should incorporate interventional experiments: (1) Targeted *in vitro* cultivation to isolate and culture key microbial hubs for direct validation of their metabolic functions; and (2) Animal intervention studies, such as fecal microbiota transplantation or supplementation.

## 5 Conclusion

This study employed an integrative multi-omics approach, combining metagenomics and untargeted metabolomics, to characterize the microbial and metabolite features of the rumen and rectum in polytocous fine-wool sheep with divergent RFI. The results demonstrated that LRFI individuals exhibit stronger homeostatic regulation and enhanced nutrient metabolic potential within local gut environments. Correlation analyses between differential microbes and metabolites, along with the constructed interaction networks, identified multiple key microbial taxa and metabolites, suggesting potential synergistic roles of microbes and metabolites in regulating RFI. Overall, this study provides mechanistic insights into microbe–metabolite interactions underlying feed efficiency, offering a theoretical foundation and potential targets for optimizing ruminant production performance.

## Data Availability

The data presented in the study are deposited in the European Nucleotide Archive (ENA) at https://www.ebi.ac.uk/ena/browser/view/PRJEB105143, accession number PRJEB105143.

## Supplementary material

SUPPLEMENTARY FIGURE S1 OPLS-DA Model parameters and permutation tests for rumen and rectum. (A) OPLS-DA score plot of rumen samples (high- vs. low-RFI). R^2^Y = 0.935, Q^2^ = 0.897. OPLS-DA score plot of rectum samples (high- vs. low-RFI). R^2^Y = 0.645, Q^2^ = 0.576. (C) Permutation test of rumen OPLS-DA model (1,000 iterations, *p* < 0.001). (D) Permutation test of rectum OPLS-DA model (1,000 iterations, *p* < 0.001).

SUPPLEMENTARY TABLE S1 Data preprocessing and quality control statistics of rumen metagenomic sequencing.

SUPPLEMENTARY TABLE S2 Data preprocessing and quality control statistics of rectal metagenomic sequencing.

SUPPLEMENTARY TABLE S3 Rumen and rectal microbial differences identified at the phylum level between the two groups.

SUPPLEMENTARY TABLE S4 Rumen and rectal microbial differences identified at the genus level between the two groups.

SUPPLEMENTARY TABLE S5 Differential microbial data identified by LEfS in the rumen.

SUPPLEMENTARY TABLE S6 Differential microbial data identified by LEfS in the rectal.

SUPPLEMENTARY TABLE S7 Differential KEGG orthologs (KOs) in the rumen with extended error bar statistics from STAMP.

SUPPLEMENTARY TABLE S8 Differential KEGG orthologs (KOs) in the rectal with extended error bar statistics from STAMP.

SUPPLEMENTARY TABLE S9 Differential metabolites in the rumen.

SUPPLEMENTARY TABLE S10 Differential metabolites in the rectal.
