# Supplementary figures and images for: Integrated multi-omics analysis reveals rumen and rectal microbiota–metabolite interaction features in polytocous fine-wool sheep with divergent residual feed intake
Source: Front Microbiol. 2026 Jan 22;16:1712307. doi: 10.3389/fmicb.2025.1712307 (PMC12880820; doi:10.3389/fmicb.2025.1712307)

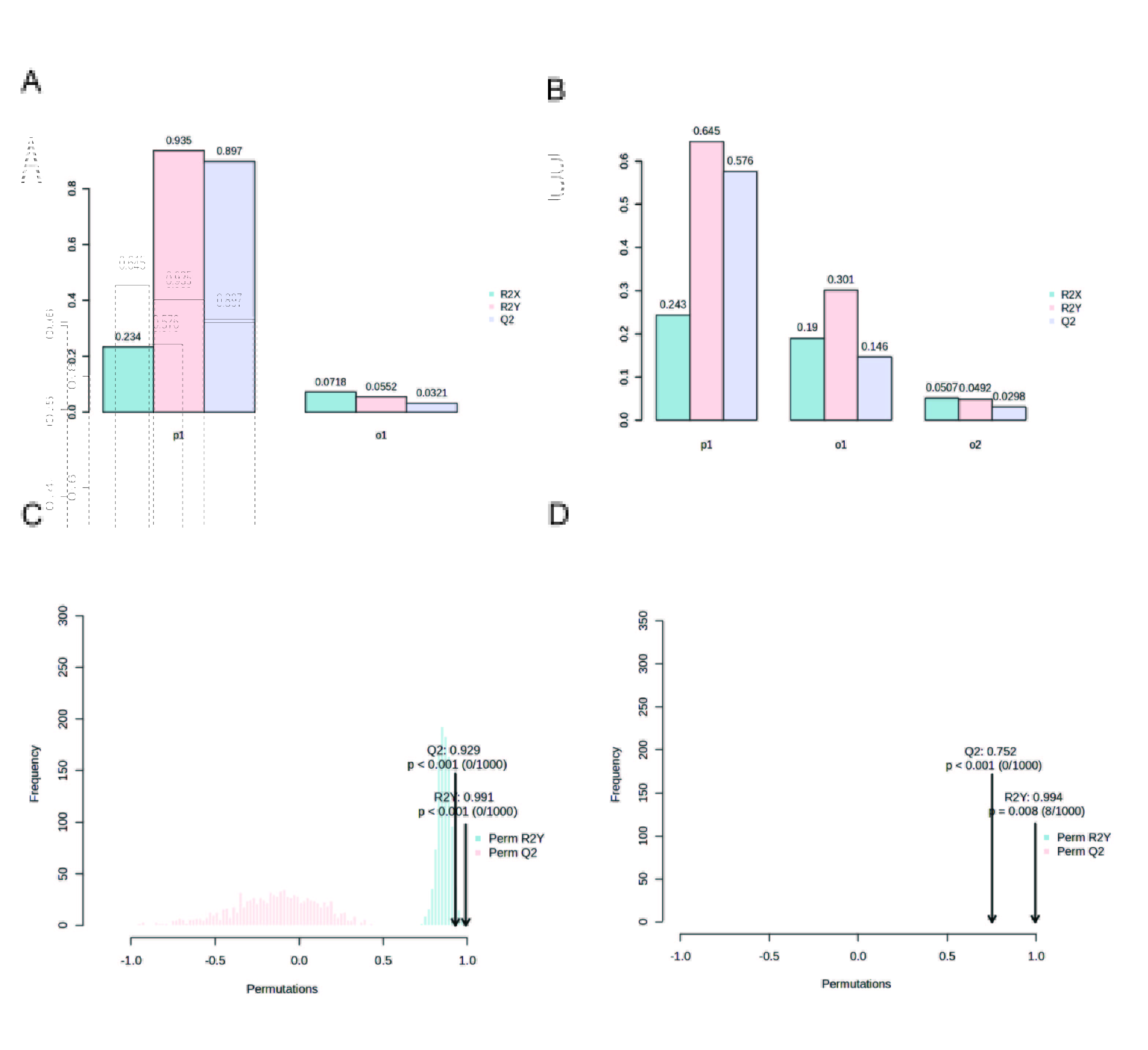

Supplement: SUPPLEMENTARY FIGURE S1 — OPLS-DA Model parameters and permutation tests for rumen and rectum. (A) OPLS-DA score plot of rumen samples (high- vs. low-RFI). R2Y = 0.935, Q2 = 0.897. OPLS-DA score plot of rectum samples (high- vs. low-RFI). R2Y = 0.645, Q2 = 0.576. (C) Permutation test of rumen OPLS-DA model (1,000 iterations, p < 0.001). (D) Permutation test of rectum OPLS-DA model (1,000 iterations, p < 0.001). [file Image_1.JPEG]
